# Metabolic Remodeling of the Parkinson’s Disease Frontal Cortex Revealed by LC-MS/MS Metabolomics

**DOI:** 10.3390/biom16060866

**Published:** 2026-06-12

**Authors:** Oluwatosin Daramola, Judith Nwaiwu, Odunayo Oluokun, Mojibola Fowowe, Alexandra Lux, Isaac Lopez, Andrew I. Bennett, Yehia Mechref

**Affiliations:** Department of Chemistry and Biochemistry, Texas Tech University, Lubbock, TX 79409, USA; odaramol@ttu.edu (O.D.); jnwaiwu@ttu.edu (J.N.); ooluokun@ttu.edu (O.O.); mfowowe@ttu.edu (M.F.); alexandra.e.lux@ttuhsc.edu (A.L.); lop90554@ttu.edu (I.L.); andy.bennett@ttu.edu (A.I.B.)

**Keywords:** metabolites, metabolomics, LC-MS/MS, Parkinson’s disease, frontal cortex

## Abstract

Parkinson’s disease (PD) is a progressive neurodegenerative disorder traditionally defined by dopaminergic neuronal loss and Lewy body pathology; however, increasing evidence indicates that metabolic dysfunction contributes to both motor and non-motor manifestations of disease. While metabolomics studies in PD have largely focused on peripheral biofluids or subcortical brain regions, metabolic remodeling within cortical regions critical for cognition remains poorly characterized. Here, we applied LC-MS/MS-based untargeted metabolomics to post-mortem frontal cortex tissue from PD and neurologically normal control donors, with statistical models adjusted for age, sex, and post-mortem interval. A total of 893 metabolites were quantified, of which 234 exhibited significant differential abundance following false discovery rate correction. Pathway enrichment and network-based integration revealed coordinated metabolic remodeling characterized by predicted inhibition of β-alanine metabolism and pantothenate-dependent coenzyme A biosynthesis alongside activation of amino acid, vitamin B-dependent, cofactor-related, redox-associated, oxidative stress, and inflammatory pathways. Recurrent alterations in pantothenic acid, β-alanine-related intermediates, arginine- and histidine-derived metabolites, lumichrome, and vitamin B_6_-associated species may reflect cortical metabolic perturbations associated with mitochondrial bioenergetic vulnerability and oxidative stress. Together, these findings indicate selective metabolic vulnerability in the PD frontal cortex rather than diffuse metabolic collapse.

## 1. Introduction

Parkinson’s disease (PD) is a progressive neurodegenerative disorder, which is known to affect approximately 1–2% of individuals over the age of 65 [1,2], and its prevalence has been recorded to rise to 4–5% in those over 85 [3]. It has also been ranked as the second most common neurodegenerative disease after Alzheimer’s disease [4]. It has primarily been clinically characterized by motor symptoms, such as bradykinesia, rigidity, resting tremors, and postural instability [5]. However, it is now widely recognized that a range of non-motor symptoms, such as olfactory impairment, gastrointestinal dysfunction, depression, cognitive decline, and autonomic disturbances, can precede motor symptoms by years [6]. PD is characterized by the selective loss of dopaminergic neurons in the substantia nigra pars compacta, as well as the abnormal accumulation of misfolded α-synuclein in Lewy bodies and Lewy neurites [7]. However, despite these defining traits, PD exhibits considerable diversity in its fundamental molecular pathophysiology and clinical presentation. This diversity suggests that multiple converging pathogenic processes, such as oxidative stress [8], mitochondrial dysfunction [9], neuroinflammation [10], and proteostasis failure [11], may drive disease progression in distinct but overlapping ways. Importantly, neurodegeneration in PD is not restricted to the basal ganglia. The frontal cortex and hippocampus are two areas of the brain that are involved in the later stages of the disease. This is linked to the cognitive impairment that most PD patients experience over time [12,13]. To fully understand the mechanism of this condition, exploring system-level approaches that include both spatial and molecular aspects is important. Although the brain constitutes just 2% of body mass, it accounts for nearly 20% of total energy consumption, reflecting its intensive demands for ionic balance, synaptic transmission, and biosynthetic activity [14]. This metabolic burden renders the brain particularly vulnerable to disruptions in energy production and metabolic homeostasis. In PD, metabolic abnormalities have been shown to be implicated in the dysfunction of proteins, lipids, and nucleic acids [15,16].

Metabolomics, the profiling of small molecules called metabolites which are less than 1.5 kDa in size, has been shown to be an effective tool for obtaining real-time biochemical states within cells and tissues [17]. In contrast to transcriptomics or proteomics, which primarily capture upstream regulatory potential or protein abundance, metabolomics provides a direct and integrative readout of ongoing physiological and pathological processes by reflecting the functional state of cellular metabolism. As such, metabolomics has been widely applied in neurological and neurodegenerative disease research to uncover biochemical alterations associated with disease onset, progression, and clinical heterogeneity. Across neurodegenerative disorders, including Alzheimer’s disease, Huntington’s disease, and amyotrophic lateral sclerosis, metabolomic studies have consistently revealed perturbations in energy metabolism, amino acid turnover, lipid homeostasis, and redox balance that are not fully captured at the transcriptomic or proteomic level [18,19,20]. Similar system-level metabolic disruptions have also been reported in experimental models of neurological and toxicological stress, as well as metabolic disorders with cognitive involvement, including traumatic brain injury, environmental neurotoxin exposure, and type 2 diabetes-associated mild cognitive impairment, further underscoring the sensitivity of metabolomics for detecting functional metabolic alterations across diverse pathological contexts [21,22,23,24].

Within PD, metabolomic investigations have identified alterations across diverse molecular classes, including amino acids, lipids, steroids, neurotransmitter-related metabolites, and redox-active species, in multiple biological matrices such as cerebrospinal fluid, plasma, and serum [1,25,26]. These findings highlight the sensitivity of metabolomics for detecting disease-associated biochemical changes and underscore its utility for biomarker discovery and mechanistic insight in PD. However, the majority of existing studies have focused on peripheral biofluids, leaving metabolic remodeling within disease-relevant cortical brain regions comparatively underexplored [27]. Despite these advances, relatively few metabolomic studies have directly interrogated human cortical brain tissue in PD, and even fewer have focused on the frontal cortex while rigorously accounting for demographic and post-mortem confounders [28]. Given the central role of the frontal cortex in cognition, executive function, and higher-order motor planning, metabolic alterations in this region may provide critical insight into the molecular substrates underlying non-motor symptoms and disease progression. Moreover, tissue-level metabolomics offers a unique opportunity to capture disease-relevant biochemical changes that may be diluted or obscured in peripheral biofluids.

Therefore, in this study, we applied LC-MS/MS-based untargeted metabolomics to post-mortem frontal cortex tissue from PD and control donors to define disease-associated metabolic alterations within a region of established clinical and pathological relevance. By integrating covariate-adjusted differential analysis with pathway enrichment, network-based modeling, and complementary knowledge-based interpretation, we sought to identify coordinated metabolic patterns rather than isolated metabolite changes. This integrative framework enables a systems-level view of PD-associated cortical metabolic remodeling, linking altered metabolite abundance to pathways involved in coenzyme A-linked energy metabolism, amino acid turnover, vitamin B-dependent and cofactor-related processes, and redox homeostasis. Collectively, this work aims to refine understanding of cortical metabolic vulnerability in PD and to provide a foundation for future mechanistic and translational investigations.

## 2. Materials and Methods

### 2.1. Ethical Statement

Post-mortem frontal cortex brain tissues from individuals with Parkinson’s disease (PD) were supplied by the Washington University Movement Disorders Center, while control tissues were obtained through the University of California San Diego Alzheimer’s Disease Research Center. All procedures were conducted in accordance with the ethical principles of the Declaration of Helsinki and were reviewed and approved by the Texas Tech University Institutional Review Board (IRB Protocol #504702; approved on 23 May 2017). Informed consent for brain donation was provided either by the donors prior to death or by their legally authorized next of kin. All tissues were fully de-identified before analysis to maintain donor confidentiality.

### 2.2. Sample Clinical Information

In this study, post-mortem frontal cortex tissues (Brodmann area 9) from 10 neurologically normal controls were obtained from the University of California San Diego Alzheimer’s Disease Research Center, while tissues from 10 individuals diagnosed with Parkinson’s disease (PD) were provided by the Washington University Movement Disorders Center. Demographic and neuropathological characteristics of all donors are summarized in Table 1.

Inclusion criteria for PD cases included a clinical diagnosis of PD during life with neuropathological confirmation of Lewy body disease at autopsy. Control cases were required to lack clinical or neuropathological evidence of PD or Lewy body disease. Cases with severe unrelated neurodegenerative pathology or insufficient tissue quality were excluded from the study. PD donors were clinically diagnosed during life and confirmed at autopsy to exhibit Lewy body disease, with Braak Lewy body stages ranging from 3 to 6, indicating limbic to neocortical involvement. Neurofibrillary tangle pathology was low (Braak NFT stages I, II), and all PD cases met criteria for Low Alzheimer’s disease neuropathologic change (ADNC) based on NIA-AA guidelines. Dementia status was documented (0 = no dementia, 1 = dementia). Control donors showed no clinical or neuropathological evidence of PD or Lewy body disease. Consistent with normal aging, control brains exhibited low or absent ADNC, with Braak NFT stages between 0 and II. Demographic characteristics including age, sex, and post-mortem interval (PMI) were recorded for all samples. The PD group had a mean age of 78.4 ± 6.1 years (range 68–86), while controls showed a mean age of 84.0 ± 7.0 years (range 73–93). Post-mortem interval (PMI) averaged 16.7 ± 12.1 h for PD donors (range 3–42 h) and 22.5 ± 20.0 h for controls (range 6–72 h). Sex distribution was comparable (PD: 6 Male/4 Female; controls: 5 Male/5 Female). These variables were examined as potential covariates in statistical analysis to account for demographic or post-mortem influences on metabolite abundance.

### 2.3. Materials

HPLC water, acetonitrile (ACN), methanol (MeOH), Dichloromethane (DCM), and formic acid (FA) were purchased from Fisher Scientific (Fair Lawn, NJ, USA). Ammonium bicarbonate (ABC) was bought from Sigma Aldrich (St. Louis, MO, USA).

### 2.4. Metabolites Extraction

Post-mortem frontal cortex tissues were obtained as frozen specimens from the respective brain repositories and stored at −80 °C until metabolite extraction and LC-MS/MS analysis. Tissue samples were carefully cut on a clean glass plate and transferred into a screw cap vial containing beads and 200 µL of 50 mM ammonium bicarbonate. The tissues were homogenized using a bead beater at 4 °C. The lysed samples were sonicated on ice for 1 h and then centrifuged at 14,800× *g* for 10 min, and the supernatants were carefully transferred into new tubes. After the protein assay, 30 µg protein was taken from each sample and made up to 50 µL with ABC buffer for metabolite extraction. The polar metabolites were extracted from 50 µL of tissue lysate by modifying the previously published method [21].

A 100 μL mixture of DCM/MeOH (1:2 *v*/*v*) was added to the samples and vortexed for 30 s. Following a 60 min incubation period at room temperature, 37.5 μL of DCM was added to the mixture, and vortexed for 30 s. An additional 37.5 μL of cold water was added and vortexed for 30 s. Finally, the samples were centrifuged for 15 min at 5000 rpm. The aqueous phase (upper layer) was then collected and transferred to new Eppendorf tubes. The samples were dried and resuspended in methanol: water (1:1), then the polar metabolites were subjected to LC-MS analysis.

### 2.5. LC-MS Analysis

The samples were separated and analyzed on an Acquity UPLC HSST3 100 Å (2.1 × 100 mm) column (Waters, Wexford, Ireland) using a Vanquish UHPLC system (Thermo Scientific, San Jose, CA, USA) coupled to a Quadrupole Exactive HF MS. Using mobile phase A comprising 0.1% FA in water and mobile phase B comprising 0.1% FA in MeOH, a multistep mobile phase gradient was used. Starting at 0.5% B, the gradient increased progressively to 50% over the next 5.5 min. The gradient was ramped up to 98% of MPB in 0.5 min and kept steady for 6 min. After that, the column was equilibrated with 0.5% MPB for 2 min.

Following the LC separation, the MS operated in both positive and negative ion modes and was used to analyze the polar metabolites using an analytical electrospray ionization source. In the positive ion mode, the spray voltage was set to 3.5 kV, and the transfer tube temperature was set to 300 °C, while 3.0 kV spray voltage was used with the temperature of the transfer tube set at 320 °C in the negative ion mode. The full MS scan was performed using an Orbitrap mass analyzer set at a mass range of 75 to 750 *m*/*z* and a resolution of 120,000. An AGC target of 3.0 × 106 and an exclusion list of the top 100 intense peaks between 0–6 min was generated from the blank that was added to the full MS method. The scan range number was configured to a value of 1, while the maximum injection duration was set to 200 ms.

To generate the MS/MS, an orbitrap scan was acquired in a data-dependent approach, employing a duty cycle of 3 s and the top four precursor ions with the highest intensity were selected for higher-energy collisional dissociation MS/MS scan. The scan was conducted using a stepped normalized collision energy of 20%, 40%, and 60% for both positive and negative ion modes. Using a Quadrupole isolation mode, with an isolation window of 4 *m*/*z* and a dynamic exclusion of 10 s for the positive ion mode, while a 1 *m*/*z* isolation window and dynamic exclusion of 6 s were set for the negative ion mode. The mass analyzer resolution was set to 30,000 for the positive mode and 15,000 for the negative mode, with a set scan range of 75 to 2000 *m*/*z*, and 50 ms maximum injection time.

### 2.6. Data Analysis

The raw data acquired was analyzed using Compound Discoverer 3.4 software to identify and quantify the metabolomics compositions present in the samples. Data cleaning, feature filtering, metabolite annotation, identifier harmonization, and pathway mapping were carried out using MetaboGraph, an in-house LC-MS/MS data processing tool developed for metabolomics and lipidomics workflows. Metabolite identities were assigned using curated database matching workflows incorporating HMDB, KEGG, PubChem, LipidMaps, and CAS identifiers together with LC-MS/MS-derived spectral annotation information. MetaboGraph’s documentation and code are shared via GitHub v1.0.1 (https://github.com/odaramola92/MetaboGraph, accessed on 2 April 2026) and on Zenodo [29]. Statistical models and all visualizations including Principal Component Analysis (PCA), Volcano plot, Venn, Box plots and Heatmaps were performed using OmicsVisStat (v1.0), a Python (version 3.13)-based platform. The underlying scripts and analysis workflows are publicly available via Zenodo [30].

Metabolite abundances were preprocessed using quality-control filters, retaining only metabolites quantified in at least 50% of samples within each cohort. Group differences between PD and control tissues were evaluated using covariate-adjusted linear regression (ANCOVA) models incorporating age, sex, and post-mortem interval (PMI) as covariates to account for demographic and technical variation. Baseline comparisons of age, PMI, and sex were evaluated using the Mann–Whitney U test and Fisher’s exact test, with Cliff’s delta calculated to quantify effect sizes. Multiple testing across metabolites was corrected using the Benjamini–Hochberg (BH) false discovery rate (FDR).

**Figure 1:**
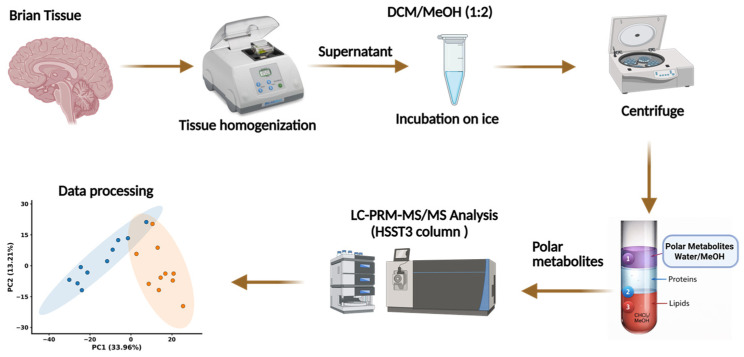
The analytical workflow for the LC-MS/MS metabolomics analysis of brain tissue.

Pathway analysis was performed using both QIAGEN Ingenuity Pathway Analysis (IPA) and MetaboGraph. MetaboGraph, as a metabolite-centric platform with extensive annotation coverage, provided Fisher-based pathway enrichment and directionality estimates, complementing IPA’s curated canonical pathway and biological function interpretation framework. All metabolite identifiers were first curated and harmonized in MetaboGraph using HMDB, KEGG, PubChem, LipidMaps, and CAS identifiers; and these validated IDs were subsequently used for IPA pathway mapping. Biological themes and pathways identified by both MetaboGraph and IPA through overlapping metabolite associations and related pathway-level interpretations were considered higher-confidence findings and prioritized in the final interpretation, whereas platform-specific observations were interpreted cautiously as complementary hypothesis-generating insights due to differences in database structure, pathway definitions, and inference methodologies between the two platforms. The analytical workflow is summarized in Figure 1.

## 3. Results

### 3.1. Donor Demographic Characteristics

Demographic comparisons between PD and control donors are summarized in Table 1. PD donors were significantly younger than controls (76.6 ± 6.2 vs. 84.0 ± 5.9 years; *p* = 0.034), with a medium effect size (Cliff’s δ = −0.57), indicating a modest but meaningful age difference between groups. Post-mortem interval (PMI) did not differ significantly (16.7 ± 12.1 vs. 22.5 ± 20.0 h; *p* = 0.818) and showed a negligible effect size (Cliff’s δ = −0.07), suggesting comparable tissue preservation conditions. Sex distribution was also similar (Fisher’s exact *p* = 0.524). As expected, all PD donors exhibited autopsy-confirmed Lewy body pathology (Braak LB stages 3–6), whereas controls showed no Lewy body disease and only age-appropriate Alzheimer-type changes. Given these patterns, age, sex, and PMI were included as covariates in subsequent statistical analyses to control for demographic and post-mortem influences on metabolite levels.

### 3.2. Metabolomics Data Overview

**Figure 2:**
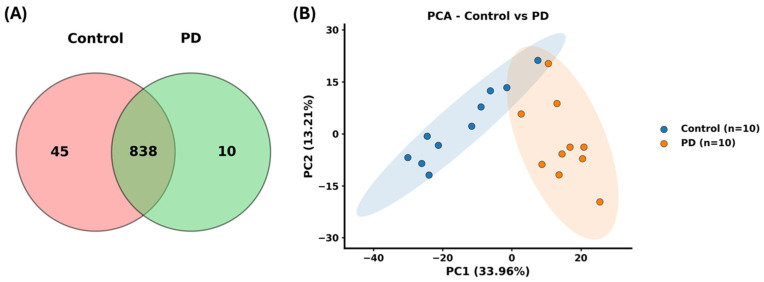
(**A**) Venn diagram showing metabolite detection overlap between control and Parkinson’s disease (PD) frontal cortex samples. The majority of metabolites (n = 838) were shared between groups, with a small number uniquely detected in controls (n = 45) or PD (n = 10), indicating largely comparable metabolite coverage across conditions. (**B**) Principal component analysis (PCA) of metabolite abundances demonstrates partial separation between control and PD samples along PC1 (33.96% variance explained), with additional variance captured by PC2 (13.21%). Ellipses represent the 95% confidence regions for each group. While some overlap remains, the overall separation suggests coordinated metabolic differences between PD and control groups at the multivariate level.

Metabolites were extracted from 20 lysed frontal-cortex brain tissues (PD = 10, Control = 10) using a DCM/MeOH mixture, followed by LC-MS/MS acquisition. Raw data were processed using Compound Discoverer to generate a feature table, which was subsequently filtered to retain only metabolites with valid structural identifiers (HMDB, KEGG, PubChem, LipidMaps, or CAS). and are listed in Supplementary Table S1, including metabolite identifiers, median log_2_-transformed abundances for each group, and corresponding statistical results. As shown in the Venn diagram in Figure 2A, 838 metabolites were consistently detected in both PD and control tissues, while 45 were unique to controls and 10 were unique to PD.

Unsupervised principal component analysis (PCA) revealed a clear separation between the PD and control groups along the first two principal components (PC1 = 33.96%, PC2 = 13.21%; Figure 2B). The clustering pattern suggests that global metabolic profiles differ between PD and non-diseased brains, supporting the presence of disease-associated metabolic alterations. Importantly, separation was not driven by extreme outliers, consistent with stable LC-MS/MS acquisition and homogeneous preprocessing. Together, the PCA and Venn analyses indicate that PD is associated with broad metabolic shifts, motivating covariate-adjusted differential analyses to identify specific dysregulated metabolites. PCA was used as an unsupervised exploratory tool to visualize global variance structure, while formal inference was performed using covariate-adjusted linear models.

### 3.3. Covariate-Adjusted Differential Metabolite Abundance Analysis

To account for demographic and post-mortem variation, group differences in metabolite abundance were assessed using linear models that included age, sex, and post-mortem interval (PMI) as covariates. Before multiple-testing correction, 279 metabolites showed nominal significance (*p* < 0.05). After applying BH correction (α = 0.05), 234 metabolites remained significantly different between PD and control groups, representing 19.8% of all metabolites tested. These results indicate that while widespread metabolic perturbations are detectable at the nominal level, a more focused set of metabolites shows robust disease-associated differences after adjusting for covariates and controlling for false discovery.

All models converged successfully and demonstrated appropriate statistical behavior, with covariates contributing variably across metabolites. Notably, age, which differed between groups, was effectively controlled in the regression model, supporting interpretation of the observed metabolite differences in the context of PD-related biology rather than demographic variation alone. Likewise, PMI and sex showed minimal independent effects but were retained to reduce residual confounding. Overall, the covariate-adjusted analysis provides a high-confidence subset of PD-associated metabolites that serve as the basis for pathway interpretation, biological contextualization, and visualization.

**Figure 3:**
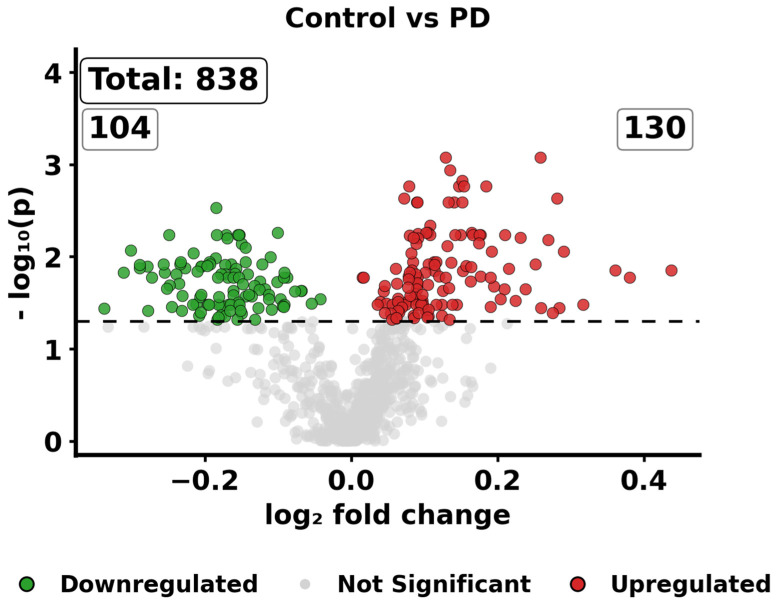
Volcano plot of all 838 metabolites following covariate adjustment for age, sex, and PMI. Metabolites significant after BH false discovery rate correction (adjusted *p*-value < 0.05) are highlighted, with metabolites increased in PD shown in red (n = 130) and metabolites decreased in PD shown in green (n = 104); non-significant metabolites are shown in gray. The dashed horizontal line indicates the adjusted *p*-value significance threshold.

The results of the covariate-adjusted differential abundance analysis were visualized using a volcano plot (Figure 3), which summarizes both effect size (log_2_ fold change) and statistical significance (−log_10_ adjusted *p*-value) for all 838 metabolites tested. Using a significance threshold (*p* < 0.05), 234 metabolites showed evidence of differential abundance between PD and control samples, comprising 130 metabolites increased and 104 metabolites decreased in PD relative to controls. Upregulated metabolites (red) exhibited positive fold changes indicative of elevated abundance in PD tissue, whereas downregulated metabolites (green) showed consistent negative fold changes, suggesting coordinated suppression of specific metabolic features.

Overall, the volcano plot indicates that PD-associated metabolic remodeling comprises a combination of directionally consistent changes and a smaller subset of metabolites with stronger effect sizes. Because significance is defined using adjusted *p*-values, the highlighted features represent high-confidence differences after controlling the false discovery rate, providing a stringent overview of disease-associated metabolic alterations in the frontal cortex.

To further examine the consistency of these alterations across individual samples, the 234 FDR-significant metabolites were visualized using unsupervised hierarchical clustering (Supplementary Figure S1). The heatmap reveals clear, group-level structure, with control and PD samples forming distinct clusters based on their metabolite abundance profiles. Metabolites increased in PD generally exhibited higher relative abundance across PD samples and lower abundance in controls, whereas metabolites decreased in PD showed the opposite pattern. Although some inter-individual variability was observed, the overall clustering pattern indicates that the majority of significant metabolites display directionally consistent changes across samples within each group. These results complement the volcano plot by demonstrating that PD-associated metabolic differences are not driven by isolated outliers but instead reflect coordinated and reproducible alterations across the cohort.

### 3.4. Pathway Analysis

To interpret the biological context of metabolites exhibiting covariate-adjusted differential abundance between PD and control frontal cortex samples, pathway enrichment analysis was performed using FDR-significant metabolites. Enrichment revealed a focused pattern of metabolic perturbation rather than diffuse pathway disruption. Vitamin- and amino acid-related processes predominated, including β-alanine metabolism, β-alanine-dependent pantothenate and coenzyme A (CoA) biosynthesis, arginine and proline metabolism, vitamin B6 metabolism, vitamin digestion and absorption, vitamin B6 activation to pyridoxal phosphate, biosynthesis of amino acids, and biosynthesis of cofactors (Supporting Figure S2). The chord-based integration in Supporting Figure S2A illustrates shared metabolite membership across enriched pathways, demonstrating substantial overlap among β-alanine, pantothenate/CoA, amino acid, and vitamin-related pathways. This overlap indicates coordinated remodeling of interconnected biochemical processes rather than isolated pathway-specific effects. In particular, β-alanine metabolism and β-alanine-dependent pantothenate and CoA biosynthesis occupy central positions within the shared-metabolite structure, consistent with mitochondrial bioenergetic vulnerability in PD cortex.

Pathway–pathway network visualization (Supporting Figure S2B) further highlights the interconnectivity of enriched pathways based on shared altered metabolites. Highly connected nodes include β-alanine metabolism, biosynthesis of cofactors, arginine and proline metabolism, vitamin B6 metabolism, vitamin digestion, vitamin B6 activation to pyridoxal phosphate, and absorption, and biosynthesis of amino acids, forming a coherent metabolic module. The dense overlap among these pathways supports a model in which PD-associated metabolic changes reflect coordinated shifts across related vitamin-dependent, amino acid, and cofactor-linked processes rather than discrete, independent perturbations.

**Figure 4:**
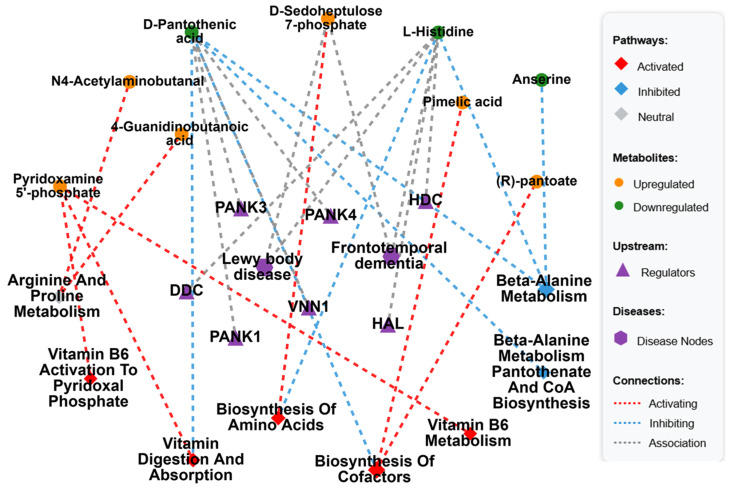
Integrated pathway network, highlighting directionally coherent metabolic regulation in PD frontal cortex. Pathways are represented as diamonds (red, activated; blue, inhibited; gray, neutral), metabolites as circles (orange, increased in PD; green, decreased in PD), upstream enzymes and transporters as triangles, and disease annotations as hexagons. Red dashed edges indicate activating relationships, blue dashed edges indicate inhibitory relationships, and gray dashed edges represent associations. Font sizes were intentionally scaled by node category to improve visual interpretation of pathway-level organization within the network.

Integration of metabolite-level changes with enzyme- and pathway-level information (Figure 4) revealed directionally coherent regulation of key metabolic pathways relevant to PD. Network analysis predicted inhibition of β-alanine metabolism and β-alanine–dependent pantothenate and CoA biosynthesis, consistent with reduced availability of pantothenic acid–derived metabolites and implicating constrained mitochondrial bioenergetic capacity. In contrast, arginine and proline metabolism, vitamin B6 metabolism, vitamin digestion, vitamin B6 activation to pyridoxal phosphate, and absorption, biosynthesis of amino acids, and biosynthesis of cofactors were predominantly activated, suggesting adaptive upregulation of amino acid turnover, vitamin-dependent enzymatic reactions, and cofactor production under conditions of metabolic stress.

The network emphasizes a focused subset of biologically relevant pathways supported by concordant metabolite-level changes rather than displaying all enriched pathways. Members of the pantothenate kinase family (PANK1, PANK3, PANK4), along with arginine- and histidine-associated enzymes and vitamin B6–dependent decarboxylases, connect altered metabolite abundances to predicted pathway regulation. Disease-associated nodes, including Lewy body disease and frontotemporal dementia, further contextualize these metabolic perturbations within established neurodegenerative frameworks. Collectively, the integrated network indicates that the PD frontal cortex exhibits coordinated metabolic remodeling characterized by suppression of CoA-linked energy pathways alongside activation of amino acid and vitamin-dependent processes, reinforcing mitochondrial vulnerability coupled with compensatory redox and metabolic adaptation.

**Figure 5:**
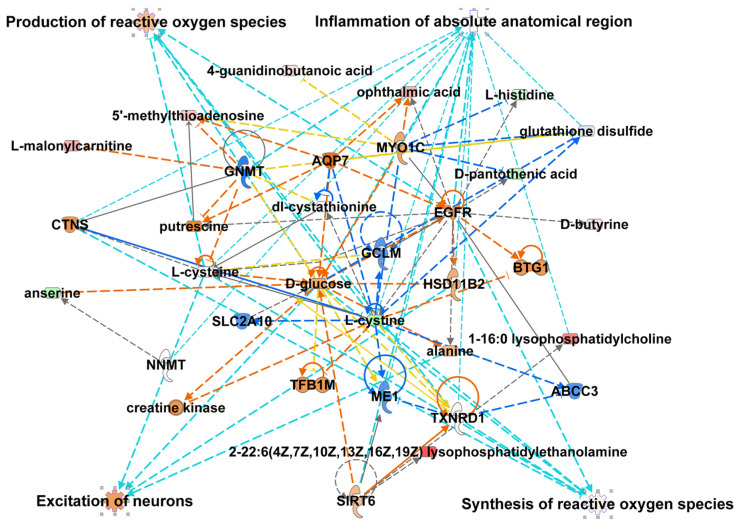
Ingenuity Pathway Analysis (IPA) network illustrating predicted biological functions and molecular interactions associated with differentially abundant metabolites in Parkinson’s disease frontal cortex. The network highlights relationships linking metabolite changes to oxidative stress, inflammation, and neuronal excitation based on curated IPA knowledge. Red and green nodes indicate increased and decreased metabolite measurements, respectively, with color intensity reflecting the relative magnitude of change. Orange and blue nodes represent predicted activation and predicted inhibition of biological functions or upstream regulators, respectively, with darker shading indicating higher prediction confidence. Edges indicate predicted relationships, including activation (orange), inhibition (blue), and findings inconsistent with the predicted downstream state (yellow).

In addition to pathway enrichment and network analysis performed using MetaboGraph, Ingenuity Pathway Analysis (IPA) was applied to provide complementary, knowledge-base-driven interpretation of the metabolomics data. All detected metabolites, along with their covariate-adjusted log_2_ fold changes and raw *p*-values, were analyzed in IPA using a nominal significance threshold (*p* < 0.05). IPA integration of metabolite abundance changes predicted activation of biological processes related to oxidative stress, neuroinflammation, and neuronal excitation, alongside modulation of glutathione metabolism and reactive oxygen species (ROS) production (Figure 5). Several metabolites and enzyme-associated nodes converged on functions associated with ROS generation and inflammatory responses, providing independent, hypothesis-generating support for metabolic dysregulation linked to established pathogenic mechanisms in PD.

**Figure 6:**
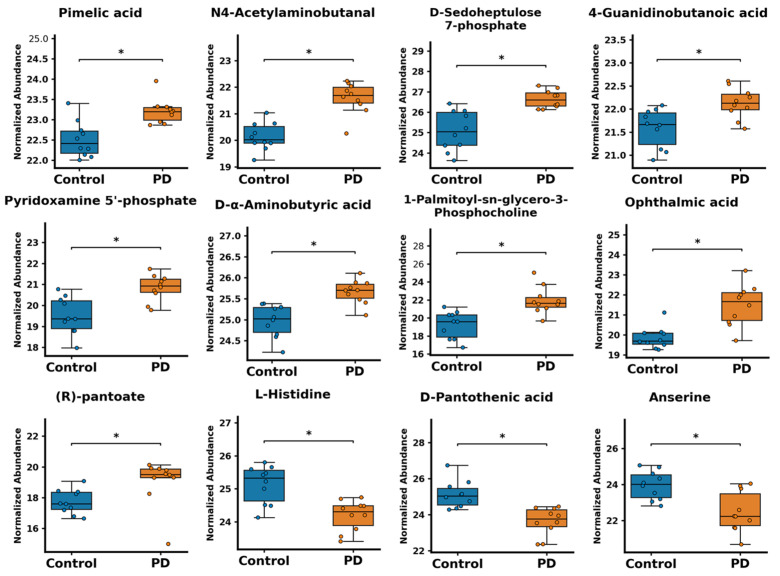
Boxplots showing representative metabolites central to the pathway perturbations identified in pathway analysis. Plots display median log_2_-transformed abundances for control and PD frontal cortex samples, highlighting metabolites involved in coenzyme A biosynthesis, amino acid metabolism, vitamin-dependent pathways, and redox processes. Statistical significance is indicated by asterisks (* *p* < 0.05).

To further illustrate how pathway-level perturbations are reflected at the individual metabolite level, metabolites contributing to key pathways identified in the pathway analysis were visualized using boxplots (Figure 6). These metabolites were selected based on their involvement in energy metabolism, amino acid and vitamin pathways, and redox-related processes highlighted in the enrichment and network analyses. The boxplots display covariate-adjusted, log_2_-transformed abundances for PD and control samples, demonstrating the direction and consistency of metabolite changes underlying the inferred pathway regulation. Notably, metabolites including Pyridoxamine 5′-phosphate, ophthalmic acid, lumichrome, D-pantothenic acid, and N4-acetylaminobutanal showed significant alterations associated with pathways linked to mitochondrial bioenergetics, oxidative stress, vitamin metabolism, and amino acid turnover.

Given the established relationship between cortical pathology and cognitive impairment in PD, exploratory analyses were additionally performed within the PD cohort to evaluate whether significant PD-associated metabolites were associated with dementia status. Correlation analyses were restricted to metabolites that were significantly altered between PD and control tissues following the primary covariate-adjusted linear modeling workflow. Although no metabolites remained significant following BH correction within the dementia subgroup analysis, a subset of biologically relevant metabolites demonstrated nominal associations (*p* < 0.05) with dementia status, including Pyridoxamine 5′-phosphate, a metabolite linked to vitamin B6 metabolism and redox-associated processes in our study. While exploratory and limited by subgroup sample size, these findings suggest that selected metabolic alterations associated with mitochondrial and vitamin-dependent pathways may also relate to cognitive manifestations of PD and warrant further investigation in larger cohorts.

## 4. Discussion

PD is a progressive neurodegenerative disorder classically defined by dopaminergic neuronal loss in the substantia nigra and the presence of Lewy body pathology [31,32]. Beyond its hallmark motor manifestations, accumulating neuropathological, imaging, and clinical evidence indicates that PD involves widespread metabolic and molecular alterations affecting cortical regions, including the frontal cortex, which contributes to cognitive, executive, and behavioral deficits, particularly in advanced disease stages [32,33,34]. Metabolic dysfunction in PD has been closely linked to mitochondrial impairment, altered neurotransmitter homeostasis, and oxidative stress, processes that are increasingly recognized as central drivers of neurodegeneration rather than secondary consequences [35,36,37]. While prior metabolomics investigations have primarily focused on biofluids or subcortical brain regions such as the substantia nigra, emerging studies suggest that cortical metabolic remodeling represents an important but underexplored aspect of PD pathology [38,39]. The frontal cortex is metabolically demanding and highly dependent on mitochondrial efficiency to support synaptic transmission, executive function, and cognitive control, making it particularly vulnerable to subtle energy deficits in PD.

In this study, we applied LC-MS/MS-based untargeted metabolomics to frontal cortex tissue from PD and control donors to characterize disease-associated metabolic alterations while controlling for demographic and post-mortem confounders. A total of 893 metabolites were quantified, of which 234 exhibited robust differential abundance following covariate adjustment and false discovery rate correction. To contextualize these changes, pathway enrichment, network-based integration, and complementary knowledge-based analysis were performed. Collectively, the results reveal directionally coherent perturbations involving predicted inhibition of β-alanine metabolism and β-alanine-dependent pantothenate and coenzyme A (CoA) biosynthesis, suppression of coenzyme A-linked energy metabolism alongside activation of amino acid, vitamins, and redox-related pathways.

At the metabolite level, a relatively focused subset of compounds emerged as central drivers of pathway-level perturbations, as evidenced by their recurrence across MetaboGraph- and IPA-derived networks. Among these, D-pantothenic acid, Pyridoxamine 5′-phosphate, L-histidine, and ophthalmic acid represent particularly informative metabolic nodes linking energy metabolism, redox balance, and mitochondrial function in the PD frontal cortex. The reduction in D-pantothenic acid observed in PD tissue is consistent with impaired CoA biosynthesis, a core requirement for mitochondrial energy metabolism, fatty acid oxidation, and acetyl-CoA availability [40,41]. Given the exceptionally high energetic demands of cortical neurons, diminished pantothenate availability may constrain mitochondrial metabolic flux and contribute to neuronal vulnerability, providing metabolite-level support for longstanding evidence of mitochondrial dysfunction in PD.

Ophthalmic acid (ophthalmate) has been associated with glutathione depletion and shifts in redox balance in metabolomic studies [42], together suggesting that PD frontal cortex exists in a state of chronic redox pressure with adaptive, but potentially insufficient, engagement of antioxidant pathways.

Consistent with this metabolite-based interpretation, pathway-level analysis revealed predicted inhibition of β-alanine metabolism and pantothenate-dependent CoA biosynthesis, implicating impaired mitochondrial bioenergetics as a central metabolic vulnerability in the PD frontal cortex. This observation aligns with prior reports of reduced pantothenic acid (vitamin B_5_) levels in multiple brain regions of PD patients, particularly in cases with dementia, suggesting constrained substrate availability for CoA production [43]. In addition, reduced levels of histidine-containing dipeptides such as anserine and carnosine have been reported in PD brain tissue and biofluids, consistent with impaired β-alanine utilization and diminished antioxidant capacity [44,45]. These findings support disruption of β-alanine-linked metabolic flux and CoA-dependent energy metabolism as contributing features of cortical metabolic dysfunction in PD. Taken together, these metabolite-centric findings suggest that PD cortical pathology is characterized not by indiscriminate metabolic collapse, but by selective vulnerability of energy-producing pathways alongside adaptive engagement of metabolite networks supporting amino acid turnover, vitamin utilization, and oxidative stress defense.

In parallel, our data reveals activation of amino acid metabolic pathways, particularly those involving arginine and proline, with additional histidine-related metabolite perturbations, suggesting adaptive responses rather than uniform metabolic failure. These findings align with a recent meta-analysis of serum amino acids in PD patients, which reported elevated proline and ornithine levels, indicative of increased arginine catabolism through the ornithine–proline axis [46]. Elevated proline may reflect perturbations in glutamate–proline cycling, while increased ornithine, a precursor for polyamine synthesis, points to altered nitrogen handling and stress-related metabolic adaptation. Consistently, untargeted metabolomics studies have identified arginine and proline metabolism among the most dysregulated pathways in PD, often alongside β-alanine metabolism [47]. Notably, reductions in carnosine and anserine, antioxidant dipeptides derived from histidine and β-alanine, have been observed in PD cortical tissue [25], further supporting altered histidine utilization and reinforcing the coordinated activation of amino acid metabolism in response to metabolic stress.

Vitamin and cofactor metabolism emerges as another compensatory axis in PD frontal cortex. We observed predicted activation of vitamin B-dependent pathways, including vitamin B_6_, consistent with prior biochemical studies of PD brain tissue reporting insufficient levels of pantothenic acid (B_5_) and pyridoxal-5′-phosphate (active vitamin B_6_) [25,43]. Epidemiological evidence further supports this association, as low dietary vitamin B_6_ intake has been linked to increased PD risk, and long-term levodopa therapy can exacerbate B_6_ deficiency due to the antagonistic effects of carbidopa [48,49]. 

The concurrent activation of cofactor biosynthesis pathways further suggests coordinated remodeling of vitamin-dependent metabolic processes in response to metabolic stress. Exploratory analyses within the PD cohort additionally suggested nominal associations between dementia status and selected metabolites linked to vitamin-dependent pathways, including Pyridoxamine 5′-phosphate, although these observations did not remain significant following multiple testing correction and should therefore be interpreted cautiously.

Consistent with these metabolic signatures, oxidative stress emerges as a convergent pathological feature in PD. Post-mortem analyses have demonstrated a ~40% depletion of reduced glutathione in PD brains, particularly within vulnerable dopaminergic regions, indicating impaired antioxidant defense early in disease progression [50]. Peripheral metabolomics findings extend this observation, with elevated serum allantoin, a marker of uric acid oxidation, reported in PD patients and reflecting heightened ROS activity [47]. Collectively, these findings suggest that PD frontal cortex exists in a state of chronic oxidative pressure, in which glutathione-centered antioxidant systems are adaptively engaged but ultimately insufficient to counteract ongoing metabolic and mitochondrial stress.

This study is associated with some limitations that should be considered when interpreting the findings. While comparable to other post-mortem human brain metabolomics studies, the modest sample size (n = 10 per group) may limit sensitivity for detecting metabolites with smaller effect sizes and may reduce the broader generalizability of the findings. Although statistical models were adjusted for age, sex, and PMI, additional factors including medication exposure, disease duration, agonal state, and comorbid conditions were not available and may contribute to residual variability in metabolite abundances. In addition, while sex was included as a covariate in the statistical models, the modest cohort size limited the ability to robustly evaluate sex-specific metabolic alterations or sex-metabolite interaction effects, which should be explored in larger future studies.

Moreover, the cross-sectional nature of the analysis and the use of end-stage tissue preclude direct inference regarding the temporal progression of metabolic alterations across disease stages. In addition, metabolite–enzyme–pathway relationships were inferred using curated biochemical databases and do not reflect direct measurements of enzymatic activity, metabolic flux, or gene expression. Consequently, pathway directionality should be interpreted as functional inference rather than direct biochemical regulation. Furthermore, the potential influence of lifetime dopaminergic therapy, including levodopa exposure, could not be comprehensively assessed because of limited clinical metadata availability and may contribute to residual metabolic variability. Finally, IPA was used to provide complementary, knowledge-base-driven biological context using nominal significance thresholds and should therefore be considered hypothesis-generating rather than confirmatory.

Future studies should extend these findings through targeted validation of key metabolic nodes identified here, particularly metabolites involved in coenzyme A biosynthesis, amino acid turnover, and vitamin-dependent redox processes. In particular, future targeted LC-MS/MS studies using authentic reference standards and quantitative validation workflows will be important to further confirm metabolite identities and accurately quantify key differential metabolites identified in this untargeted analysis. Longitudinal analyses across earlier disease stages and additional cortical and subcortical regions would help clarify whether the observed metabolic signatures represent early pathogenic drivers or later compensatory responses. Integration with transcriptomic, proteomic, and flux-based approaches in future multi-omics studies may further refine mechanistic interpretation, identify coordinated molecular alterations associated with PD pathology, and resolve enzyme-level regulation underlying metabolite changes. Finally, translation of these findings to biofluids may facilitate the development of minimally invasive biomarkers reflective of cortical metabolic dysfunction in PD.

## 5. Conclusions

In summary, this LC-MS/MS-based metabolomics study reveals that PD frontal cortex is characterized by coordinated metabolic remodeling rather than diffuse metabolic disruption. By integrating covariate-adjusted differential analysis with pathway enrichment, network-based integration, and complementary knowledge-based interpretation, we identify a consistent pattern marked by predicted inhibition of β-alanine metabolism and pantothenate/coenzyme A (CoA) biosynthesis alongside activation of amino acid, vitamin B-dependent, and cofactor-related pathways. These directionally coherent alterations are consistent with mitochondrial bioenergetic vulnerability accompanied by compensatory metabolic adaptations aimed at sustaining neurotransmitter synthesis and redox homeostasis. The convergence and network-level integration of metabolite-level involving changes on β-alanine/pantothenate metabolism, amino acid turnover, vitamin B-dependent pathways, and oxidative stress responses provides a mechanistic framework linking cortical metabolic dysfunction to established features of PD pathology. Together, these findings expand current understanding of PD beyond classical nigrostriatal degeneration, highlighting the frontal cortex as a site of metabolically encoded vulnerability and adaptation. This work establishes a foundation for future studies aimed at validating key metabolic nodes, resolving temporal dynamics across disease stages, and translating cortical metabolic signatures into accessible biomarkers and therapeutic targets.

## Figures and Tables

**Table 1:** Demographic, clinical, and neuropathological characteristics of the PD and control cohorts.

Demographics	PD	Control	*p*-Value	Cliff’s Delta (δ)
Age (years)	76.6 ± 6.2 (range: 68–86)	84.0 ± 5.9 (range: 73–93)	0.034	−0.57
Sex (Male/Female)	six/four	five/five	0.524	-
Post-mortem Interval (PMI, hours)	16.7 ± 12.1 (range: 3–42)	22.5 ± 20.0 (range: 6–72)	0.818	−0.07
Clinical Dementia Status (0 = none, 1 = dementia)	9/1	10/0	-	-
Lewy Body Pathology (Braak LB Stage)	3–6	0	-	-
Neurofibrillary Tangle Stage (Braak NFT Stage)	I–II	0–II	-	-
Aβ Amyloid Stage (A0–A3)	A1–A2	A0–A2	-	-
CERAD Neuritic Plaque Score (C0–C3)	C0–C1	C0–C1	-	-
NIA–AA ADNC Classification	Low ADNC	Not AD/Low ADNC	-	-
Other Neuropathology	Mild small vessel disease; occasional ARTAG; rare lacunes	Mild age-related vascular changes	-	-

## Data Availability

The mass spectrometry data has been deposited to massive with permanent URL ftp://massive-ftp.ucsd.edu/v12/MSV000100628/, accessed on 2 April 2026. Review URL access: ftp://MSV000100628@massive-ftp.ucsd.edu, accessed on 2 April 2026. Password: PD_Metabolite.

## Supplementary Materials

The following supporting information can be downloaded at: https://www.mdpi.com/article/10.3390/biom16060866/s1, Figure S1: Heatmap of the 234 metabolites significantly altered between Parkinson’s disease (PD) and control frontal cortex samples following covariate adjustment and FDR correction (adjusted *p* < 0.05). Data were log_2_-transformed, scaled by metabolite, and hierarchically clustered to visualize group-level abundance patterns. Red and green indicate higher and lower relative abundance, respectively; Figure S2: Metabolite-pathway Chord diagram and pathway-pathway overlap analysis of PD-associated metabolic alterations. (A) Metabolite–pathway chord diagram illustrating relationships between FDR-significant metabolites and enriched metabolic pathways in PD versus control frontal cortex samples. Pathways are arranged circumferentially, and connecting ribbons represent shared metabolite membership between metabolites and pathways. Ribbon color encodes the direction and magnitude of metabolite change (green, decreased in PD; red, increased in PD). (B) Pathway-pathway network constructed based on shared metabolite membership among enriched pathways. Nodes represent metabolic pathways and are colored according to enrichment significance (*p*-value), as indicated in the legend. Edges denote pathway associations derived from shared altered metabolites, with greater connectivity reflecting increased metabolite overlap; Table S1: List of all metabolites with structural identifiers, median log_2_-transformed abundances for PD and control samples, and corresponding statistical results (Annexed as an Excel file).

## Abbreviations

The following abbreviations are used in this manuscript:

PD	Parkinson’s disease
LC-MS/MS	Liquid chromatography-Tandem mass spectrometry
PCA	Principal component analysis
PMI	Post-mortem interval
BH	Benjamini–Hochberg
FDR	False discovery rate
CoA	Coenzyme A
GSH	Glutathione
ROS	Reactive oxygen species
IPA	Ingenuity pathway analysis
NFT	Neurofibrillary Tangle
ADNC	Alzheimer’s Disease Neuropathologic Change
NIA-AA	National Institute on Aging-Alzheimer’s Association
