# Supplementary material for: Metabolic Remodeling of the Parkinson’s Disease Frontal Cortex Revealed by LC-MS/MS Metabolomics
Source: Biomolecules. 2026 Jun 12;16(6):866. doi: 10.3390/biom16060866 (PMC13296450; doi:10.3390/biom16060866)
Supplement: Supplementary file 1 [file biomolecules-16-00866-s001.zip › Metabolomics_SI_R2.pdf]

[Supplementary data]

Metabolic Remodeling of the Parkinson's Disease Frontal Cortex Revealed by LC-MS/MS Metabolomics

Oluwatosin Daramola, Judith Nwaiwu, Odunayo Oluokun, Mojibola Fowowe, Alexandra Lux, Isaac Lopez, Andrew I. Bennett, and Yehia Mechref*

Department of Chemistry and Biochemistry, Texas Tech University, Lubbock, TX 79409-1061

***Corresponding Author**

Department of Chemistry and Biochemistry

Texas Tech University

Lubbock, TX 79409-1061

Email: yehia.mechref@ttu.edu

Tel: 806-742-3059

Fax: 806-742-1289

Table of Contents

List of Figures

Supplementary Figure S1. Heatmap of the 234 metabolites significantly altered between Parkinson's disease (PD) and control frontal cortex samples following covariate adjustment and FDR correction (adjusted $p < 0.05$). Data were $\log_2$-transformed, scaled by metabolite, and hierarchically clustered to visualize group-level abundance patterns. Red and green indicate higher and lower relative abundance, respectively.

Supporting Figure S2. Metabolite-pathway Chord diagram and pathway-pathway overlap analysis of PD-associated metabolic alterations. (A) Metabolite–pathway chord diagram illustrating relationships between FDR-significant metabolites and enriched metabolic pathways in PD versus control frontal cortex samples. Pathways are arranged circumferentially, and connecting ribbons represent shared metabolite membership between metabolites and pathways. Ribbon color encodes the direction and magnitude of metabolite change (green, decreased in PD; red, increased in PD). (B) Pathway-pathway network constructed based on shared metabolite membership among enriched pathways. Nodes represent metabolic pathways and are colored according to enrichment significance (p-value), as indicated in the legend. Edges denote pathway associations derived from shared altered metabolites, with greater connectivity reflecting increased metabolite overlap.

List of Tables

Supplementary Table S1: List of all metabolites with structural identifiers, median $\log_2$-transformed abundances for PD and control samples, and corresponding statistical results (Annexed as an Excel file).

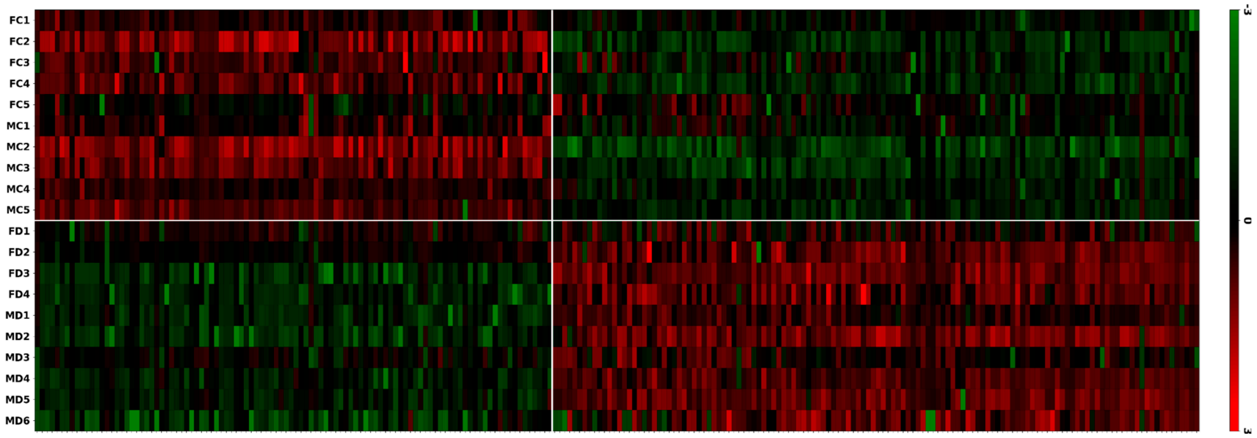

Supplementary Figure S1. Heatmap of the 234 metabolites significantly altered between Parkinson's disease (PD) and control frontal cortex samples following covariate adjustment and FDR correction (adjusted $p < 0.05$). Data were log_2-transformed, scaled by metabolite, and hierarchically clustered to visualize group-level abundance patterns. Red and green indicate higher and lower relative abundance, respectively.

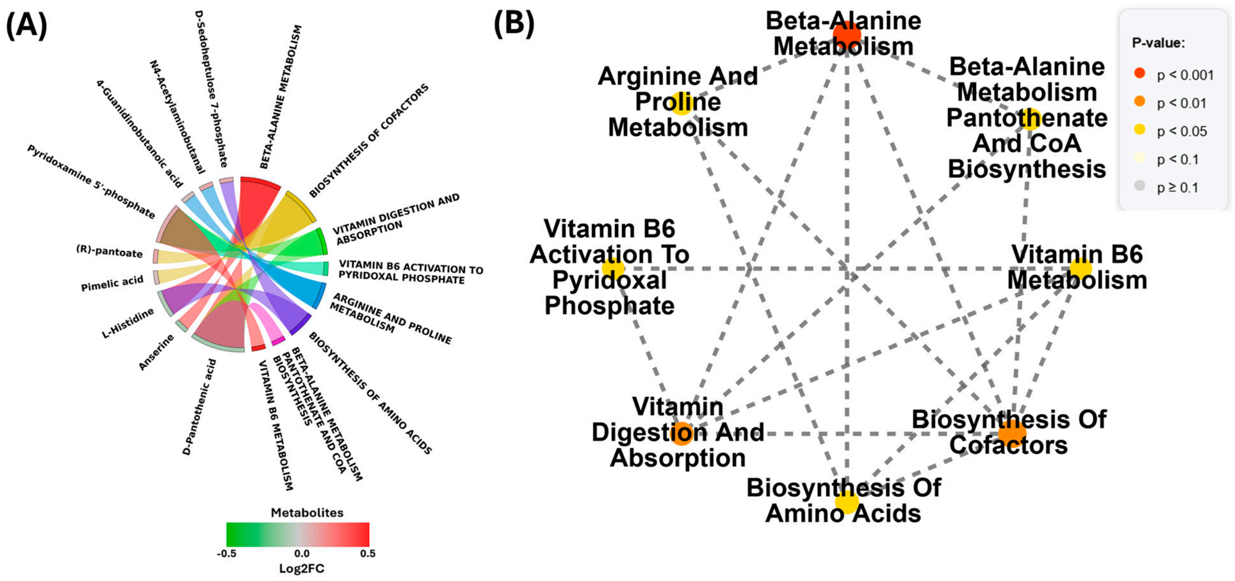

Supporting Figure S2. Metabolite-pathway Chord diagram and pathway-pathway overlap analysis of PD-associated metabolic alterations. (A) Metabolite–pathway chord diagram illustrating relationships between FDR-significant metabolites and enriched metabolic pathways in PD versus control frontal cortex samples. Pathways are arranged circumferentially, and connecting ribbons represent shared metabolite membership between metabolites and pathways. Ribbon color encodes the direction and magnitude of metabolite change (green, decreased in PD; red, increased in PD). (B) Pathway-pathway network constructed based on shared metabolite membership among enriched pathways. Nodes represent metabolic pathways and are colored according to enrichment significance (p-value), as indicated in the legend. Edges denote pathway associations derived from shared altered metabolites, with greater connectivity reflecting increased metabolite overlap.
